# Enhancement of NAD^+^-dependent SIRT1 deacetylase activity by methylselenocysteine resets the circadian clock in carcinogen-treated mammary epithelial cells

**DOI:** 10.18632/oncotarget.6002

**Published:** 2015-10-26

**Authors:** Mingzhu Fang, Wei-Ren Guo, Youngil Park, Hwan-Goo Kang, Helmut Zarbl

**Affiliations:** ^1^ Robert Wood Johnson Medical School, Rutgers, The State University of New Jersey, Piscataway, NJ, USA; ^2^ School of Public Health, Rutgers, The State University of New Jersey, Piscataway, NJ, USA; ^3^ Environmental and Occupational Health Sciences Institute, Rutgers, The State University of New Jersey, Piscataway, NJ, USA; ^4^ NIEHS Center for Environmental Exposures and Disease, Rutgers, The State University of New Jersey, Piscataway, NJ, USA; ^5^ Cancer Institute of New Jersey, Rutgers, The State University of New Jersey, Piscataway, NJ, USA; ^6^ Veterinary Drugs & Biologics Division, Animal and Plant Quarantine Agency, Anyang 430-757, Republic of Korea

**Keywords:** circadian clock, *N*-methyl-*N*-nitrosourea, methylselenocysteine, period 2, SIRT1

## Abstract

We previously reported that dietary methylselenocysteine (MSC) inhibits *N*-methyl-*N*-nitrosourea (NMU)-induced mammary tumorigenesis by resetting circadian gene expression disrupted by the carcinogen at the early stage of tumorigenesis. To investigate the underlying mechanism, we developed a circadian reporter system comprised of human mammary epithelial cells with a luciferase reporter driven by the promoter of human *PERIOD 2* (*PER2*), a core circadian gene. In this *in vitro* model, NMU disrupted cellular circadian rhythm in a pattern similar to that observed with SIRT1-specific inhibitors; in contrast, MSC restored the circadian rhythms disrupted by NMU and protected against SIRT1 inhibitors. Moreover, NMU inhibited intracellular NAD^+^/NADH ratio and reduced NAD^+^-dependent SIRT1 activity in a dose-dependent manner, while MSC restored NAD^+^/NADH and SIRT1 activity in the NMU-treated cells, indicating that the NAD^+^-SIRT1 pathway was targeted by NMU and MSC. In rat mammary tissue, a carcinogenic dose of NMU also disrupted NAD^+^/NADH oscillations and decreased SIRT1 activity; dietary MSC restored NAD^+^/NADH oscillations and increased SIRT1 activity in the mammary glands of NMU-treated rats. MSC-induced SIRT1 activity was correlated with decreased acetylation of BMAL1 and increased acetylation of histone 3 lysine 9 at the *Per2* promoter E-Box in mammary tissue. Changes in SIRT1 activity were temporally correlated with loss or restoration of rhythmic *Per2* mRNA expression in NMU-treated or MSC-rescued rat mammary glands, respectively. Together with our previous findings, these results suggest that enhancement of NAD^+^-dependent SIRT1 activity contributes to the chemopreventive efficacy of MSC by restoring epigenetic regulation of circadian gene expression at early stages of mammary tumorigenesis.

## INTRODUCTION

The circadian clock regulates a wide range of cellular and physiological processes in a precise and sustained rhythm with a periodicity of ~24 hrs. The clock comprises molecular oscillators functioning in both the central pacemaker (suprachiasmatic nucleus, SCN) and the cells composing most peripheral tissues. In mammalian cells, the periodicity of circadian clock is regulated by interconnected transcriptional/translational feedback loops. Heterodimers of circadian transcription factors, BMAL1 (Brain-Muscle Arnt-Like protein 1) and either CLOCK or NPAS2, regulate transcription by binding to E-box elements in the promoters of core circadian genes (CGs) [e.g., *Period* (*Per) gene*], and numerous circadian-controlled genes (CCGs) (e.g., hormone receptors, growth associated genes and DNA damage response and repair genes) [1]. As circadian proteins accumulate in the cell, they are post-translationally modified and transported to the nucleus to repress or activate Clock:Bmal1 transcriptional activity. In this way, core circadian genes limit their own transcription and set up the rhythmic expression of CGs and CCGs [2]. These intrinsic molecular oscillators can be reset by external signals including light, genotoxic stress, nutrients, hormones, and environmental signals [1–4]. Thus, circadian clocks integrate a wide variety of environmental and cellular inputs to maintain normal cellular and physiological homeostasis under changing conditions.

One of the important functions of circadian clock is to regulate the organism's response to genotoxic stress (e.g., carcinogen exposure). The circadian clock regulates the transcription, translation, and post-translational modification of ~10% genes involved in DNA damage response and repair and cell-cycle progression [5]. Circadian rhythm also can be reset or disrupted by genotoxic agents, increasing the susceptibility of cells to DNA damage and carcinogenesis [6–8]. As a result, disruption of circadian rhythm by lifestyle, occupational, and genetic factors has been associated with an increased risk of various types of cancers, including breast cancer [9, 10].

The promoting effect of circadian disruption on carcinogenesis is consistent with the finding that Per2 has tumor suppressing activity [2]. Knocking out or mutating *Per2* increases cancer cell growth, and accelerates spontaneous and carcinogen-induced tumor development in rodents. By contrast, normal or ectopic expression of clock genes induces cell cycle arrest and sensitizes cancer cells to DNA damage-induced apoptosis after exposure to genotoxic stress. Decreased expression of circadian genes is observed in various human cancers, including breast cancer, and genetic variants, mutations, and epigenetic modifications of CGs are associated with invasive and aggressive breast cancer [2, 11, 12]. In addition, *Per2* links the circadian cycle to estrogen receptor signaling [13, 14]. These findings indicate that maintenance of normal rhythmic expression of CGs (e.g., *Per2*) plays an important role in suppression of mammary tumorigenesis.

Restoration of normal circadian rhythm has been linked to chemopreventive activity of dietary organic selenium (Se) in our recent studies [13, 15]. Selenium is a trace element essential to numerous biological processes, including antioxidant defense systems, thyroid hormone metabolism, and immune function [16]. Both epidemiology and animal studies have revealed that selenium compounds have chemopreventive activity against various cancers, including breast cancer. In particular, *L*-methyl-selenocysteine (MSC) and its metabolites showed the greatest inhibitory effect on mammary and prostate tumorigenesis, especially during early stages, in rodent models [16, 17]. Furthermore, MSC also improves therapeutic efficacy and ameliorates systemic toxicity of anticancer drugs in various animal models [18, 19]. Multiple mechanisms have been proposed to explain selenium-mediated chemoprevention; however, many unsuccessful clinical trials indicated that more mechanistic studies are needed for development of effective intervention strategies [20]. Recent *in vitro* studies showed that selenium compounds protect against peroxynitrite-induced DNA damage through glutathione-mediated redox cycling [21]. MSC induces cell senescence through activation of the ATM-mediated DNA repair upon exposure to genotoxic and oxidative stresses [22]. The latter effects are supported by clinical intervention studies showing that selenium supplementation reduced oxidative DNA damage and cancer incidence in carriers of *BRCA1* mutations [23]. Moreover, selenium modulates multiple transcription factors activities involved in cellular responses to stress, including NFĸB [24], p53 [25], and BMAL1 [18]. Selenium compounds also up-regulate tumor suppressor gene expression by inhibiting the activity of histone deacetylases (HDACs) [26] and DNA methyltransferases [27].

Our previous studies demonstrated that *in vivo* exposure to carcinogen disrupts the circadian clock of target tissues. Our studies were the first to link chemopreventive efficacy of organic selenium to restoration of circadian clock in carcinogen-exposed rats [13, 15]. Exposure to a single carcinogenic dose of *N*-nitroso-*N*-methylurea (NMU) resulted in an apparent disruption in the rhythmic expression of CGs and CCGs in the mammary glands of female Fisher rats at day 30 post-exposure. Dietary MSC, given at chemopreventive dose for 30 days beginning after carcinogen exposure, reduced the incidence of mammary adenocarcinoma by ~60% [15, 28]. MSC also reset and enhanced the rhythmic expression of CGs, especially *Per2*, and CCGs, including several growth-regulatory genes and DNA damage responsive and repair genes [13, 15]. In the present study, we investigated the mechanisms by which MSC is able to reset and to enhance CG expression disrupted by the carcinogen. The protracted time course and reversibility of NMU-initiated effects by MSC suggested that both NMU and MSC might alter epigenetic regulation of circadian gene expression [29]. Consistent with this possibility, previous studies implicated phosphorylation of histone 3 (H3) at serine 10 as a regulator of circadian gene expression [30]. Further studies documented rhythmic acetylation of H3 on circadian gene promoters, with a peak acetylation corresponding with peak transcription [31]. The discovery that the circadian transcription factor, CLOCK, has intrinsic histone acetyltransferase activity further implicated chromatin remodeling in regulation of CGs and CCGs [32]. The NAD^+^-dependent deacetylase, Sirtuin 1 (SIRT1) counterbalances CLOCK-directed acetylation of its hetero-dimerization partner, BMAL1 at lysine 537, and histone 3 at lysine 9 (H3K9) [33]. Genetic ablation or pharmacological inhibition of SIRT1 activity disturbs circadian acetylation of H3 and BMAL1 [33]. Given that the SIRT1 activity is regulated by intracellular NAD^+^/NADH, we investigated the effects of NMU and MSC on the NAD^+^-SIRT1 pathway and their impact on mammary circadian clock using both *in vitro* and *in vivo* models.

## RESULTS

### MSC restored cellular circadian rhythms disrupted by NMU and SIRT1 inhibitors in mammary epithelial cells *in vitro*

We previously reported that dietary MSC inhibits NMU-induced mammary tumorigenesis by resetting circadian gene expression disrupted by the carcinogen at early stages of tumorigenesis. To investigate the underlying mechanisms, we developed a circadian reporter system comprising human mammary epithelial cells with a destabilized firefly luciferase reporter driven by the promoter of human *PER2*. The *PER2* promoter was selected as a surrogate for circadian gene expression in our *in vitro* system, because it has been demonstrated in our previous *in vivo* studies to be the major clock gene linking the circadian rhythm and chemoprevention and it is known to be directly regulated by the circadian transcription factor, BMAL1:CLOCK. Using this reporter system, we were able to monitor the cellular circadian rhythm of gene expression *in vitro* for couple of days after synchronization.

**Figure 1:**
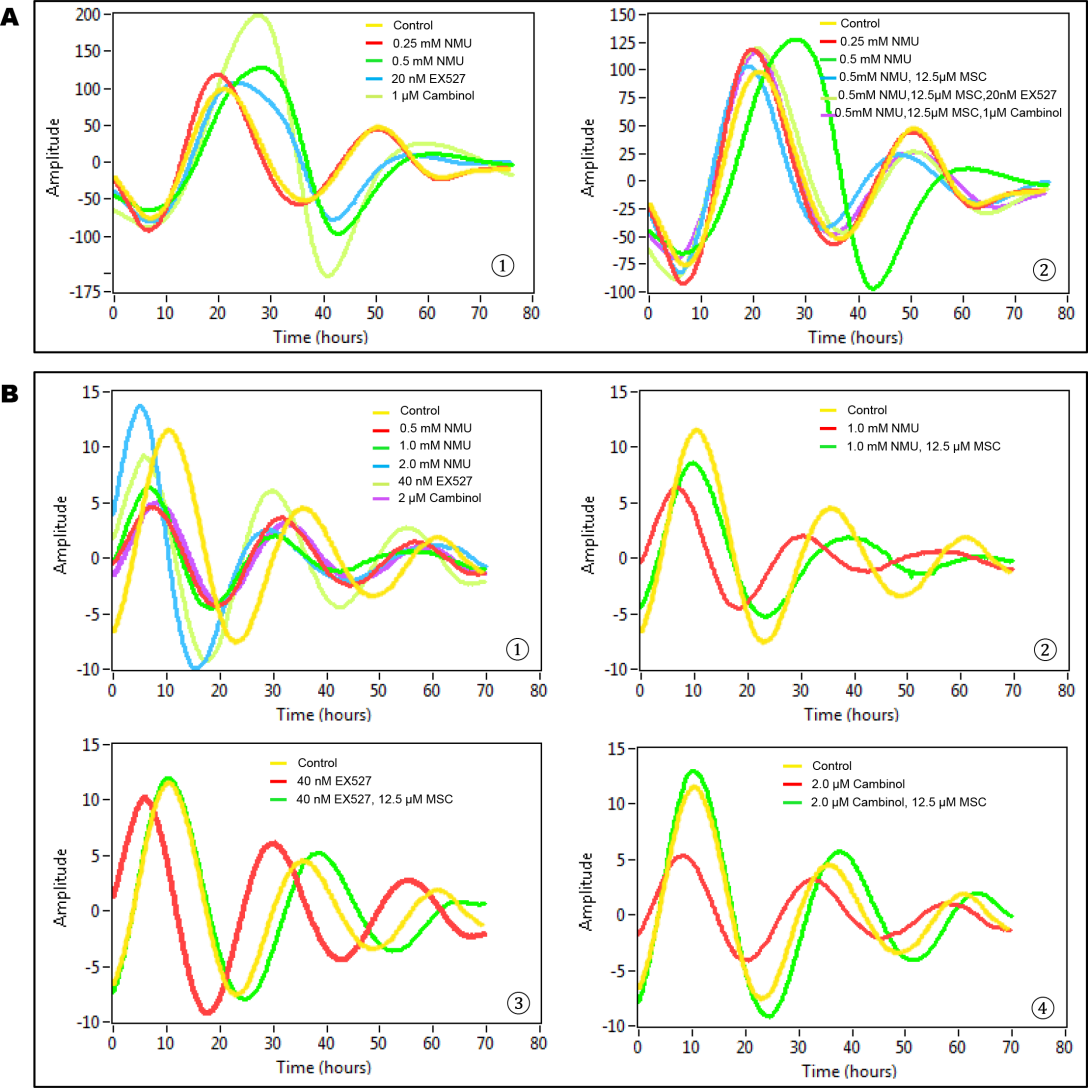
MSC restored cellular circadian rhythms disrupted by NMU and SIRT1 inhibitors in mammary epithelial cells *in vitro* Bioluminescence assays were performed on MCF10A/*PER2-dLuc* reporter cells after synchronization with 50% horse serum. X-axis, time (hours) (post-NMU treatment time); Y-axis, amplitude. **A.** Results from transiently transfected cells. ➀ Cells were treated with 0, 0.25 or 0.5 mM NMU, 20 nM EX527, or 1 μM cambinol for 1 hour following synchronization. Yellow: control; red: 0.25 mM NMU; green: 0.5 mM NMU; blue: 20 nM EX527; yellow green: 1 μM cambinol. ➁ Cells were treated with 12.5 μM MSC alone, or in combination with 20 nM EX527 or 1 μM cambinol in recording medium following exposure to 0.5 mM NMU. Yellow: Control; red: 0.25 mM NMU; green: 0.5 mM NMU; blue: 0.5 mM NMU + 12.5 μM MSC; yellow green: 0.5 mM NMU + 12.5 μM MSC + 20 nM EX527; purple: 0.5 mM NMU + 12.5 μM MSC + 1 μM cambinol. **B.** Results from stably transfected cells. ➀ Cells were treated with 0, 0.5, 1.0 or 2.0 mM NMU, 40 nM EX527, or 2 μM cambinol for 1 hour after synchronization. Yellow: Control; red: 0.5 mM NMU; green: 1.0 mM NMU; blue: 2.0 mM NMU; yellow green: 40 nM EX527; purple: 2 μM cambinol. ➁ Cells were treated with 12.5 μM MSC in recording medium following exposure to 1.0 mM NMU. Yellow: Control; red: 1.0 mM NMU; green: 1.0 mM NMU + 12.5 μM MSC. ➂ Cells were treated with 12.5 μM MSC following exposure to 40 nM EX257. Yellow: Control; red: 40 nM EX257; green: 40 nM EX257 + 12.5 μM MSC. ➃ Cells were treated with 12.5 μM MSC following exposure to 2 μM cambinol. Yellow: Control; red: 2 μM cambinol; green: 2 μM cambinol + 25 μM MSC.

Untreated, transiently transfected cells (control group) showed two complete cycles of luminescence signaling after synchronization (Fig. 1A). Treatment with 0.25 mM NMU did not disrupt the cellular circadian rhythm. Exposure to 0.5 mM NMU initially delayed and later abolished circadian rhythm, as indicated by disappearance of the second peak of luminescence at ~50 hours post-treatment. Inhibition of SIRT1 activity with 20 nM Ex257 (SIRT1 specific inhibitor) or 1 μM cambinol (SIRT1 and SIRT2 dual inhibitor) similarly disrupted circadian rhythm by dampening the subsequent circadian cycle (Fig. 1A➀). Importantly, addition of MSC (12.5 μM) into culture medium restored circadian rhythm in NMU-treated cells towards the normal in both first and second cycles. MSC not only restored the rhythm disrupted by NMU but also prevented the disruptive effects of SIRT1 inhibitors, Ex257 and cambinol (Fig. 1A➁).

In stably transfected cell lines, both NMU and SIRT1 inhibitors disrupted circadian rhythm, albeit at higher concentrations than that observed in transient transfectants (Fig. 1B➀). MSC (12.5 μM) restored circadian rhythms in the cells pretreated with 1 mM NMU (Fig. 1B➁). More importantly, MSC also restored circadian rhythms in the cells treated with SIRT1 inhibitors, including EX257 (40 nM) (Fig. 1B➂) and cambinol (2 μM) (Fig. 1B➃), respectively.

### MSC counteracts NMU to restore SIRT1 activity and NAD^+^/NADH ratio in mammary epithelial cells *in vitro*

**Figure 2:**
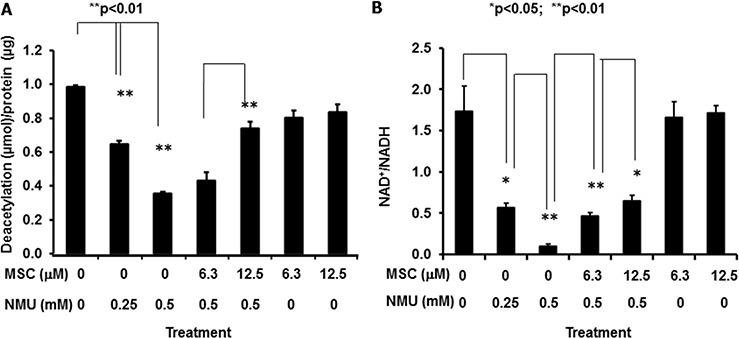
MSC counteracts NMU to restore SIRT1 activity and NAD^+^/NADH in mammary epithelial cells *in vitro* MCF10A cells were treated with NMU for 1 hour, followed by treatment with MSC for 72 hours at indicated concentrations. **A.** Total protein samples extracted from cells were used in determination of SIRT1 activity. X-axis: treatment group; Y-axis: SIRT1 activity, shown as deacetylated product (μmol)/protein (μg), mean ± SE (*n* = 3). **B.** Cell extracts were prepared and used in NAD^+^/NADH quantification. X-axis: treatment group; Y-axis: NAD^+^/NADH, mean ± SE (*n* = 3). * And ** indicate statistical significance at *p* ≤ 0.05 and *p* ≤ 0.01, respectively.

In order to test our hypothesis that NMU and MSC modulate the mammary circadian clock by altering NAD^+^-SIRT1 pathway in mammary epithelial cells, we further determined the SIRT1 activity in the human mammary epithelial cells after treatment with MSC for 72 hours following exposure to NMU for 1 hour. Our results indicated that NMU induced a dose-dependent decrease in SIRT1 activity, while addition of MSC at the concentration (12.5 μM) that can restore the cellular circadian rhythm disrupted by NMU or SIRT1 inhibitors, significantly increased SIRT1 activity in the cells treated with 0.5 mM NMU, without a significant effect on the untreated control cells (Fig. 2A). These results suggested that the inhibitory effect of NMU on SIRT1 activity may be at least partially responsible for the disappearance of the second peak of cellular circadian rhythm in the reporter cells, while induction of SIRT1 activity by MSC rescued the second cycle of rhythm disrupted by NMU (Fig. 1). These findings demonstrate that NMU and MSC alter cellular circadian rhythm by modulating SIRT1 activity. However, increasing doses of either NMU (0–1 mM) or MSC (0–25 μM) showed no significant effect on purified recombinant SIRT1 enzyme activity *ex vivo* (data not shown), indicating that neither compound directly modulates SIRT1 protein deaceylase activity.

We further observed that the intracellular NAD^+^/NADH ratio was decreased as a function of increasing dose (0–0.5 mM) of NMU. MSC significantly increased NAD^+^/NADH in a dose-dependent manner in the cells pretreated with NMU, but not in untreated control cells (Fig. 2B). These results indicate that NMU and MSC modulate SIRT1 activity by changing NAD^+^/NADH in mammary epithelial cells.

### MSC restored the NMU-disrupted circadian expression of *Per2* mRNA in rat mammary glands *in vivo*

**Figure 3:**
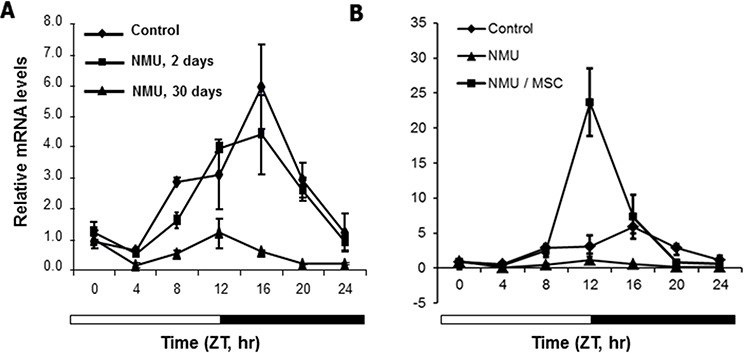
MSC restored the NMU-disrupted circadian expression of *Per2* mRNA in mammary glands of NMU-treated rats *in vivo* Three rats in each group were sacrificed every 4 hours over a 24-hour period, beginning at 7 AM. *Per2* mRNA expression levels were determined using RT-qPCR with total RNA samples extracted from rat mammary glands, and the results were analyzed with a comparative Ct method. Results were normalized with endogenous control, *β-actin*, and then with the expression level of the first sample at ZT0. X-axis: Zeitgeber Time (ZT), empty bar indicates light-on from ZT0 (7 AM) and black bar indicates light-off from ZT12 (7 PM); Y-axis: relative mRNA level, shown in mean ± SE (*n* = 3). **A.** NMU-treated rats were sacrificed at day 0 (control, diamond), 2 (square), or 30 (triangle) post-treatment. **B.** NMU-treated rats were maintained on control diet (triangle) or MSC-enriched diet (square) for 30 days post-exposure to NMU; rats untreated and maintained on control diet were used as control group (diamond).

To test whether the effects of NMU and MSC on cellular circadian rhythm *in vitro* can also be observed *in vivo*, we examined the rhythmic expression of *Per2* in mammary glands of pubertal female rats at day 2 or 30 following exposed to NMU and maintained on MSC-enriched diet or control diet. An apparent circadian rhythm of *Per2* mRNA expression was observed in mammary glands of control rats. Exposure to a single carcinogenic dose of NMU induced a gradual and persistent, time-dependent decrease in the rhythmic expression of *Per2* mRNA in mammary glands of rats. The slight disruptive effect of NMU on the rhythmic expression of *Per2* mRNA was first detectable two days after exposure, and it progressed to complete disruption by day 30 (Fig. 3A). A dietary supplement of MSC given for 30-days after NMU not only prevented and/or reversed the disruptive effects of NMU on circadian rhythm, but also significantly enhanced the rhythmic expression of *Per2* (Fig. 3B). These results recapitulated our *in vitro* results and reproduced our previous observation *in vivo* [13].

### MSC reset the circadian rhythm of NAD^+^/NADH and increased SIRT1 activity in mammary glands of NMU-treated rats *in vivo*

**Figure 4:**
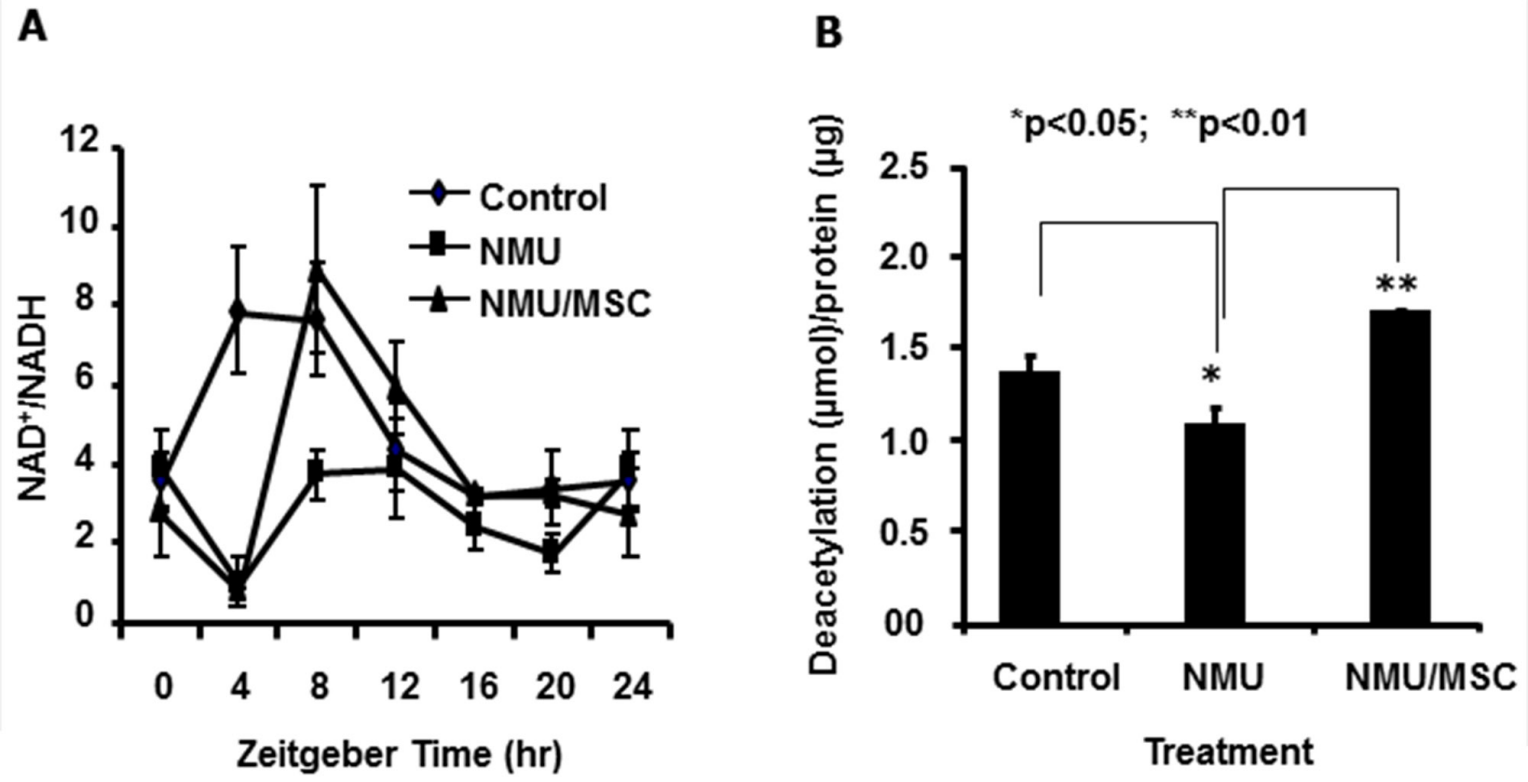
MSC reset the circadian oscillation of NAD^+^/NADH and increased SIRT1 activity in mammary glands of NMU-treated rats *in vivo* On day 30 post-exposure to NMU, three rats per group (Control, NMU, or NMU/MSC) were sacrificed every 4 hours over a 24 hour period, beginning right after lights-on at 7 AM (ZT0). **A.** Tissue extracts prepared from mammary tissues of rats sacrificed at different time points over 24 hours subjected to NAD^+^/NADH quantification. X-axis: Zeitgeber time (ZT); Y-axis: NAD^+^/NADH, mean ± SE (*n* = 3). **B.** Total protein samples extracted from mammary tissues of rats sacrificed at ZT12 subjected to SIRT1 activity determination. X-axis: treatment group; Y-axis: SIRT1 activity, shown as deacetylated product (μmol)/protein (μg), mean ± SE (*n* = 3). * And ** indicate statistical significance at *p* ≤ 0.05 and *p* ≤ 0.01, respectively.

Next, we examined if the effects of NMU and MSC on NAD^+^/NADH and SIRT1 activity that we observed *in vitro* also happened in rat mammary tissue *in vivo* during mammary tumorigenesis. NAD^+^/NADH in mammary glands of control rats showed a normal circadian pattern over a 24-hour period, with a peak at ZT4–8. At day 30 after exposure to NMU, the circadian oscillation in the NAD^+^/NADH was ablated as compared to the control. However, dietary MSC restored both the rhythm and the levels of NAD^+^/NADH to those seen in control rats, and reset the circadian phase, with the peak at ZT8 (Fig. 4A). Consistent with decreased levels of NAD^+^/NADH, mammary glands of NMU-treated rats showed decreased SIRT1 activity as compared to control rats. By contrast, SIRT1 activity was significantly higher in mammary glands of NMU-exposed rats maintained on MSC-enriched diet (Fig. 4B). Importantly, the results from the *in vivo* studies were consistent with the findings in our *in vitro* studies. These results connect the effect of MSC on NAD^+^-dependent SIRT1 activity in the present study to its previously reported chemopreventive activity *via* the restoration of circadian expression of *Per2* [13].

### MSC counteracts the effects of NMU on acetylation of BMAL1 and H3K9 associated with *Per2* gene promoter in rat mammary glands *in vivo*

**Figure 5:**
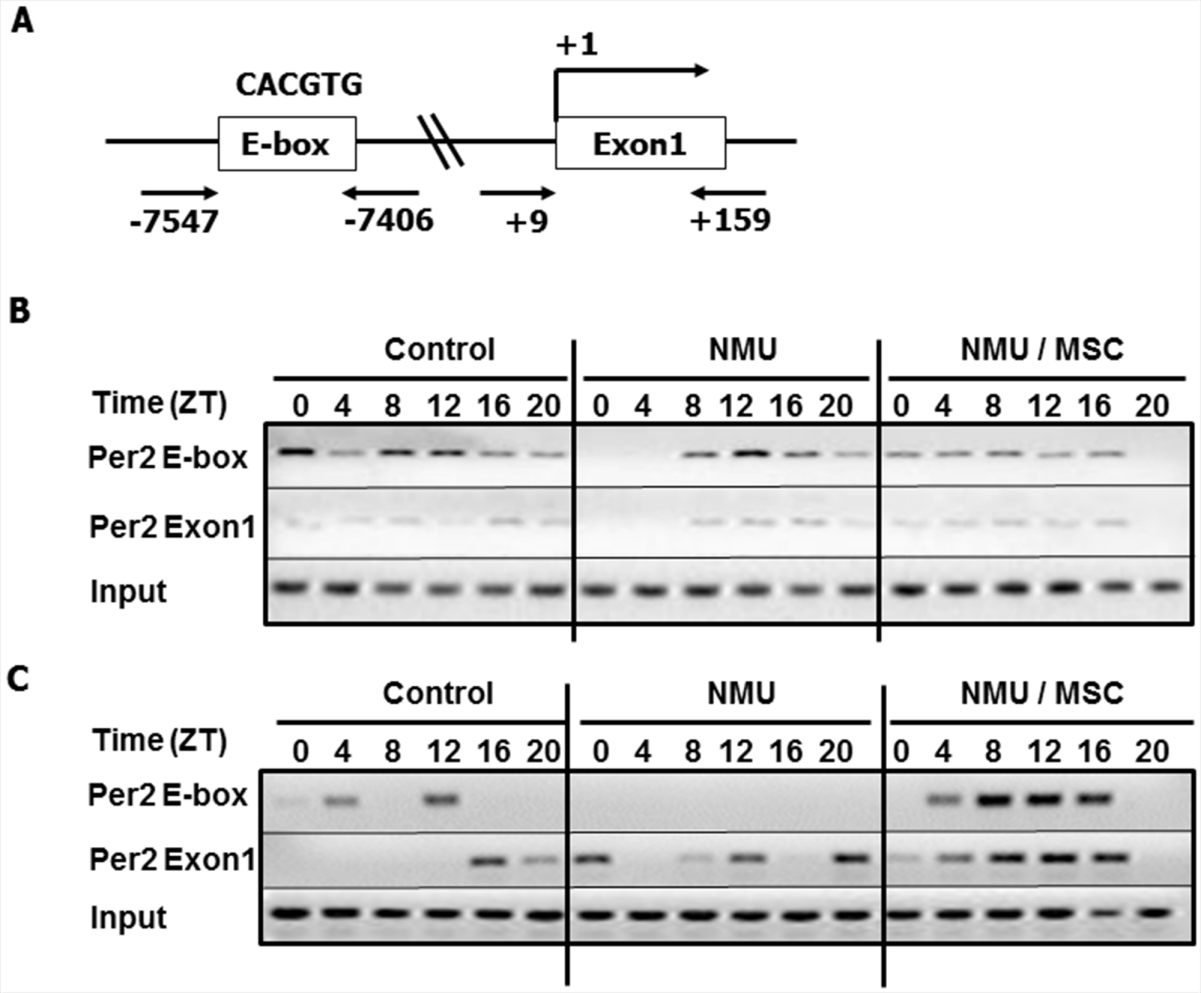
Dietary MSC counteracted the effects of NMU on acetylation of BMAL1 and H3K9 in *Per2* promoter in mammary glands of NMU-treated rats *in vivo* ChIP assay was performed with anti-AcBMAL1 or anti-AcH3K9 antibody and specific primer set against *Per2* E-box or Exon1 in *Per2* promoter. On day 30 post-exposure, three rats in each group were sacrificed every 4 hours over 24 hour, beginning at 7 AM (ZT0). Pooled mammary tissues from three rats per time per group subjected to ChIP. **A.** Schematic representation of the *Per2* promoter illustrates the regions of PCR amplicons against E-box binding motif and Exon1 in ChIP; **B.** AcBMAL1 levels; **C.** AcH3K9 levels. Upper, E-box; middle, Exon1; lower, input control.

To further investigate the mechanisms by which the NMU- and MSC-induced alterations in SIRT1 activity modulate circadian gene expression, we used a Chromatin Immunoprecepitation (ChIP) assay to evaluate their effects on acetylation of BMAL1 and histones at E-box and Exon 1 within the *Per2* promoter *in vivo* (Fig. 5A). The analysis showed that AcBMAL1 bound preferentially to the E-box relative to Exon 1 in *Per2* promoter, indicating that circadian transcription of *Per2* is associated with AcBMAL1 at *Per2* promoter E-Box in mammary glands, as reported for other organs [32]. In controls, AcBMAL1 binding to the *Per2* promoter was higher during the day (ZT0-12) than at night (ZT16-20), with the Per2 expression peaking at ZT16 (Fig. 5B & Fig. 3). At day 30 post-exposure to NMU, we observed that binding of AcBMAL1 was delayed by 4–8 hours, with peak binding at ZT12. Importantly, MSC advanced the phase of AcBMAL1 binding by 4–8 hours to match that of control cells, albeit with reduced binding, especially at ZT12-20 compared to that in NMU group (Fig. 5B). These observations indicated that MSC counteracts the effect of NMU on AcBMAL1 at E-box to induce *Per2* mRNA expression *in vivo* (Fig. 5B & Fig. 3).

Since acetylation status of histones was shown to be associated with the circadian transcription of *Per2* and the acetylation process is also under circadian control [32], we next examined acetylation of histone 3 at lysine 9 (AcH3K9) on the E-box and Exon1 of the *Per2* promoter after exposure to NMU and MSC. ChIP assay results showed that in controls, the peak of highest AcH3K9 level at E-box occurred at ZT12, four hours prior to the peak in *Per2* mRNA; peak AcH3K9 level at Exon1 occurred at ZT16, corresponding to the peak *Per2* mRNA transcription. In NMU-treated rats on control diet, there was no detectable AcH3K9 at the E-box and no rhythmic expression of *Per2* mRNA expression; AcH3K9 was detectable at Exon1 although the change in level did not result in rhythmic pattern of *Per2* mRNA expression. By comparison, NMU-treated rats maintained on MSC-enriched diet showed diurnal changes in AcH3K9 at both Exon1 and E-box sites, with peak values at ZT 8-12 (Fig. 5C & Fig. 3). These results suggest that circadian pattern of AcH3K9 at the E-box is temporally correlated with the rhythm, while AcH3K9 at Exon1 is correlated with the change of level in *Per2* mRNA expression (Fig. 5C & Fig. 3). While NMU completely abolished AcH3K9 at E-box and circadian rhythm of *Per2* mRNA expression, MSC-enriched diet restored circadian patterns of AcH3K9 in promoter regions and enhanced rhythmic mRNA expression of *Per2* in NMU treated animals.

## DISCUSSION

Both epidemiology and animal studies have indicated that selenium reduces the incidence of various cancers, stimulating several large-scale intervention trials using various organic selenium compounds [16, 34]. Unfortunately, none of these interventions showed protective effects of selenium compounds, highlighting the need for defining and matching the mechanistic underpinnings with compounds that target patients with specific exposures and/or risk factors. The present study investigated the mechanism by which MSC restores circadian gene expression, with the goal of using mechanistic insights to develop intervention strategies to reduce the risk of breast cancer associated with exposures that disturb circadian rhythm.

A wide array of metabolic and physiological processes display daily oscillations [35, 36], and an interplay exists between circadian clocks and metabolic rhythms in all organisms [37]. Metabolic rhythms connect the circadian clock via cycles of epigenetic modification. Two of these, the NAD^+^ and acetylation cycles, affect the amplitude of circadian gene expression [32]. Correlation between DNA damage-associated NAD^+^ depletion and decrease of SIRT1 activity in older rats suggested that NAD^+^ is important for cancer prevention and longevity [38]. The repair of single-strand DNA breaks by base excision is facilitated by poly-(ADP-ribose) polymerase (PARP), which uses NAD^+^ as a substrate. Since NAD^+^ also serves as an important redox carrier to power oxidative phosphorylation and ATP production, prolonged activation of PARP depletes NAD^+^ and causes cell death [39].

We hypothesize that exposure to DNA-damaging agents depletes intracellular NAD^+^ and decreases SIRT1 activity, attenuating circadian rhythm, reducing the ATM/CHEK2-mediated DNA repair, which in turn activates PARP to further deplete NAD^+^. NMU is a potent alkylating agent. Base excision repair is the principal mechanism by which mammalian cells repair alkylated DNA. Depleting of cellular NAD^+^ is therefore a plausible mechanism by which NMU could ablate epigenetic regulation of circadian genes. Excision repair–induced depletion of NAD^+^ could decrease SIRT1 activity, leading to increased AcBMAL1 at the corresponding time point and repressed circadian expression of *Per2* and CCGs, including DNA repair genes and tumor suppressor genes [13]. This hypothesis is supported by our observation from both *in vitro* and *in vivo* assays. Mammary cell death and inflammation after NMU exposure could also contribute to the disruption of circadian rhythm *in vivo* at the early time point [40], but are unlikely to precipitate the observed progressive and persistent circadian disruption over 30 days.

Selenium compounds protect cells against peroxynitrite-induced DNA damage through glutathione-mediated redox cycling and enhance cellular defenses against oxidative DNA damage [21]. Accordingly, selenium supplementation decreased oxidative DNA damage and reduced breast cancer risk in recent intervention trials [23]. The selenium-modulated cycling of GSH/GSSG is coupled to NADP^+^/NADPH and NAD^+^/NADH, suggesting that selenium affects levels of NAD^+^ metabolites through its antioxidant properties [21]. We found that MSC regulates redox cycling (NAD^+^/NADH), increasing NAD^+^-dependent SIRT1 activity and expression of the major CGs, *Per2*. Since the expression of ~10% of genes involved in DNA repair and cell proliferation is controlled by circadian rhythm [2], MSC-induced circadian expression might enhance ATM/CHEK2-mediated DNA repair to reduce DNA damage and prevent NAD^+^ depletion. Selenium compounds have also been shown to activate ATM-mediated repair in response to genotoxic and oxidative stresses, which could contribute to prevent NAD^+^ depletion and reset circadian rhythm, enhancing barriers to tumorigenisis [22].

Further mechanistic studies indicated that the MSC-induced increase of SIRT1 activity correlated temporally with decreased AcBMAL1 at the E-box element of *Per2* promoter. These changes correlated with enhanced circadian *Per2* transcription, supporting the contention that SIRT1 is required for circadian transcription of major CGs (*e.g., Per2)*. These findings are consistent with previous studies showing that SIRT1 uses NAD^+^ to remove acetyl groups from BMAL1, decreasing AcBMAL1 and leading to enhanced CLOCK/BMAL1 binding to E-boxes, acetylation of H3 and transcription of *Per2* [33, 41]. Alternatively, enhanced deacetylation and degradation of PER2 protein by increased SIRT1 activity could also be a mechanism underlying the restoration of rhythmic *Per2* mRNA expression [42]. Additional studies will be required to fully understand the mechanism by which the reduced (or enhanced) NAD^+^-SIRT1 activity caused disruption (or restoration) of rhythmic expression of *Per2* mRNA in mammary epithelial cells.

Significantly, we observed similar results in mammary cells both *in vitro* and *in vivo*. By contrast, *in vitro* studies in mouse embryo fibroblasts indicated that SIRT1 inhibitors actually enhanced the rhythmic expression of the *Dbp* gene during the circadian first cycle post serum shock [33]. We detected a similar effect when mammary epithelial cells were treated with NMU or SIRT1 inhibitors *in vitro*. These observations suggest that the initial response to agents that perturb circadian regulation may be induction of compensatory mechanisms. However, our *in vitro* results indicated that despite this initial adaptive response, cells treated with NMU or SIRT1 inhibitors immediately showed dampening of subsequent circadian cycles. The inability to reestablish a second cycle suggests that imbalance in the circadian clock machinery compromises amplitude control and/or the cells were unable to sustain the initial compensatory response [43]. In the case of genotoxicants, this could be due to depletion of intracellular NAD^+^ by DNA repair processes. Increase of NAD^+^/NADH by MSC could prevent depletion of intracellular NAD^+^, allowing the circadian gene expression to persist in subsequent cycles. Nonetheless, further investigations are needed to address these differences.

It is important to note that the regulation of circadian gene expression can vary significantly among cell types, species and even strains of mice [44, 45]. Of particular note is that regulation of circadian gene expression is independent of melatonin in many mouse strains [45]. The absence of the melatonin-mediated circadian control leads to a compensatory substitution of energy balance/food intake as a mediator of circadian regulation [46]. As a result, the primary circadian regulatory mechanisms and responses to stressors may show commensurate variations.

**Figure 6:**
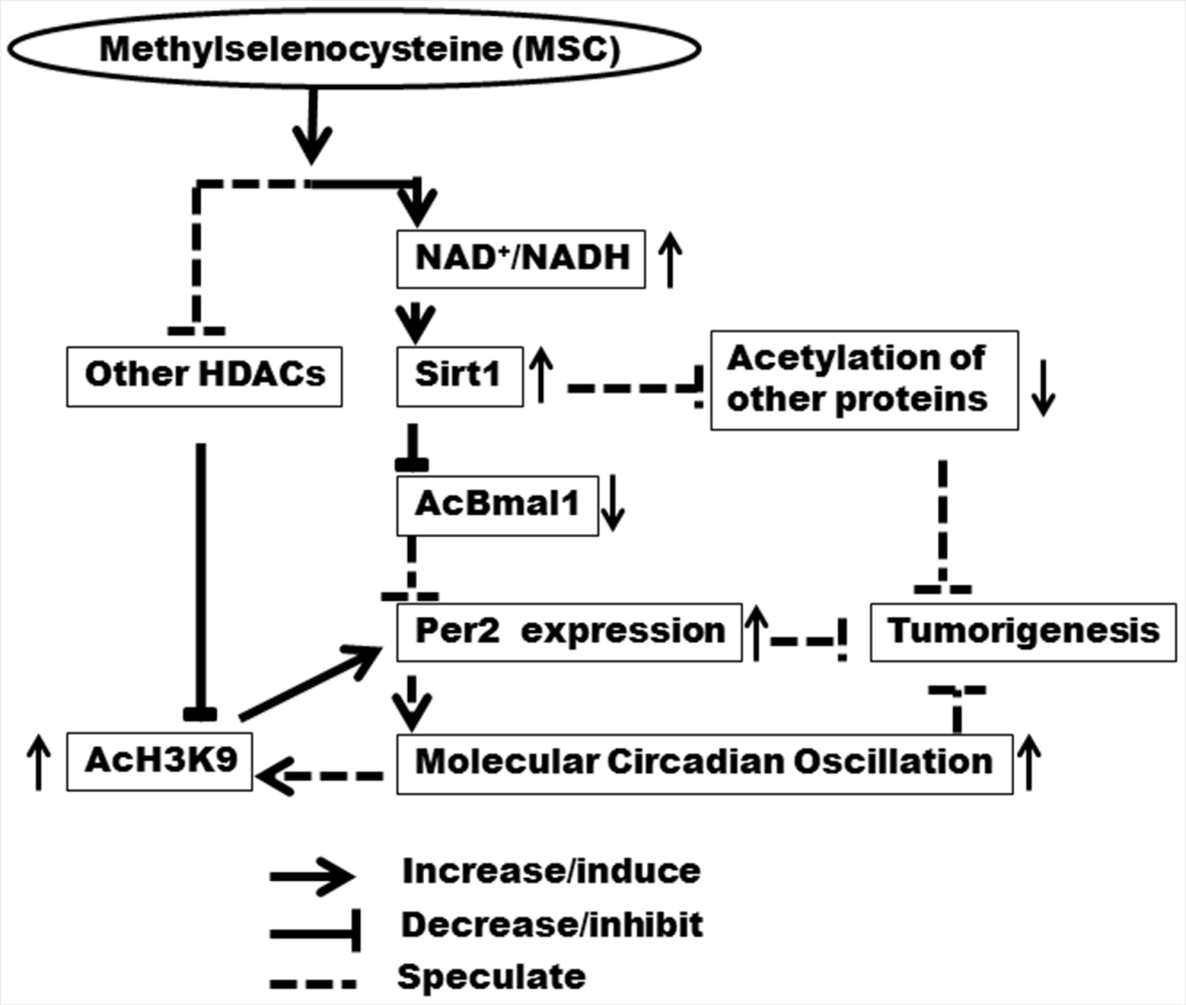
Mechanistic diagram of NAD^+^/NADH and SIRT1 in regulation of circadian rhythm by MSC Enhancement of NAD^+^-dependent SIRT1 activity by MSC restores circadian rhythm *via* restoring epigenetic regulation of *Per2* expression, which may contribute to chemopreventive activity of MSC at the early stage of tumorigenesis.

In our studies, MSC-induced SIRT1 activity was correlated with restoration and enhancement of circadian histone acetylation (AcH3K9). These findings are consistent with previous studies showing that inhibition of SIRT1 activity *in vitro* abolished rhythm of AcK3K9 at the E-box motif of the circadian outcome gene, *Dbp* [33]. In addition, the rhythmic AcH3K9 not only regulates the rhythmic mRNA expression of *Per2* gene, but is also controlled by the circadian clock [31]. The enhanced AcH3K9 rhythm might thus be regulated by increased circadian rhythm that was modulated by decreased-AcBMAL1 resulting from increased NAD^+^-dependent SIRT1 activity by MSC. Alternatively, the inhibitory effect of MSC on other histone deacetylases could also contribute to the increased level of AcH3K9 *in vivo* [26]. These results suggest that enhancement of NAD^+^-dependent SIRT1 activity by MSC can restore circadian gene expression following carcinogen exposure. The resulting increase in molecular circadian oscillation may resynchronize the AcH3K9 cycling in the promoters of CGs and CCGs to further amplify the rhythmic gene expression (Fig. 6). Regardless of a precise molecular mechanism, our observation on the ability of selenium to modulate circadian transcriptional activity is supported by a previous report indicating that selenium modulates the circadian clock to protect mice from the toxicity of a chemotherapeutic drug [18].

Together with our previous findings in chemoprevention studies [13, 15], these results indicate that MSC enhances NAD^+^/NADH oscillation and SIRT1 activity to increase circadian expression of genes involved in DNA repair and tumor suppression, contributing to early barrier to tumorigenesis. Although the role of SIRT1 gene in carcinogenesis remains controversial [47], studies in various *in vitro* studies and *in vivo* models have found a significant association between the increases in NAD^+^ level and SIRT1 activity by fasting, exercise, and other chemopreventive regimens and the prevention of aging-related diseases, including cancer and metabolic syndrome [48–50]. Increased SIRT1 activity may prevent the early stage pathogenesis by restoring or enhancing circadian rhythms. These findings suggest that the ability of various SIRT1 activating compounds, including plant polyphenols, niacin vitamins, and nicotinamide riboside, to prevent stress-related disease processes may be partially mediated through their effects on circadian rhythm [51]. The ability of dietary MSC to modulate circadian rhythm by enhancing SIRT1 activity may have therefore significant implication in chemoprevention of cancer and other aging-related chronic diseases. Testing of a large panel of cell lines and animal models would be needed to determine how universal the restoration effect of dietary MSC on circadian rhythm disrupted by environmental factors is. Further studies are warranted to assess whether the chemopreventive potential of MSC can be enhanced when used in combination with other agents that prevent NAD^+^ depletion and activate SIRT1 activity during the early stage of tumorigenesis.

## MATERIALS AND METHODS

### Cell culture and treatment

Immortalized, non-tumorigenic human mammary epithelial cell line (MCF10A) was purchased from the American Type Culture Collection (ATCC), where cells were cytogenetically tested and authenticated with short tandem repeat analysis before freezing. Each vial of frozen cells was thawed and maintained in culture for a maximum of 8 weeks. Cells were cultured in mammary epithelial cell growth medium (MEGM) (Lonza), containing mammary epithelial basal medium (MEBM) and growth supplements provided in SigleQuots Kit (Lonza) (including 0.4% bovine pituitary extract, 0.1% insulin, 0.1% hydrocortisone, 0.1% human epidermal growth factors, and 0.05% gentamycin sulfate and 0.05% amphotericin-B). Cholera toxin (Sigma) was also added to MEGM at a final concentration of 100 ng/ml. Cells were incubated at 37°C, 95% humidity and 5% CO_2_.

For NAD^+^/NADH and SIRT1 activity assays, cells were treated with NMU (0.25 or 0.50 mM) or vehicle control (0.1% DMSO) for 1 hour at 37°C. After washing with 1XPBS, cells were treated with MSC (6.3 or 12.5 μM) or vehicle control (0.1% ethanol) for 72 hours.

### Cellular circadian rhythm assay

A fragment (947 bp) of the promoter region of human *PER2* (*hPER2P*) containing 3 of BMAL1 binding motifs (CAC/TGTG) was obtained from a plasmid, pLS[hPER2P/rLuc/Puro] (LightSwitch Genomics). *hPER2P* was subcloned into destabilized firefly luciferase (dLuc) vector with neomycin selection gene [pGL(Luc2P/Neo)] (Promega), producing a pGL[hPER2P(Luc2P/Neo)] construct. MCF10A cells were seeded in 35-mm dishes at 2 × 10^5^ cells per dish and transfected with 1 μg of vector using FuGene HD™ transfection reagent (LightSwitch Genomics). After 48 hours post-transfection, cells were starved from growth factors in MEBM for 24 hours. After synchronization with 50% horse serum (HS) (Invitrogen) for 2 hours, cells were treated with 0.25 mM or 0.5 mM NMU, 20 nM EX527 (a SIRT1-specific inhibitor, Sigma), or 1 μM cambinol (inhibit both SIRT1 and SIRT2, Sigma) for 1 hour in MEBM. After washing with PBS, recording medium [MEBM supplemented with 20% of normal concentrations of growth supplements, 6.5 mM sodium bicarbonate, 10 mM HEPES buffer (pH 7.2), 0.1 mM Luciferin (Promega), and 50 units/ml penicillin and 50 μg/ml streptomycin] containing 12.5 μM MSC alone, or in combination with 20 nM EX527 or 1 μM cambinol was added. Plates were sealed with a sterile glass cover slide using silicon grease and subjected to continuous monitoring by an LumiCycle 32™ (Actimetric). Stably transfected MCF10A/*PER2-dLuc* cells selected with 1000 μg/ml of G418 (Gibco) were treated with NMU at 0.5, 1, or 2 mM, EX527 at 40 nM, or Cambinol at 2.0 μM for 1 hour after synchronization with 50% HS, and then the cells were incubated in recording medium containing 12.5 μM of MSC and monitored by LumiCycle for 4–5 days. By using LumiCycle Analysis Software (Actimerics), the data were detrended (running average) and then best-fits to a sine wave were estimated by a Levenberg-Marquardt algorithm for measurement of period, phase, amplitude, and damping rate as reported previously [52]. All treatment concentrations used were lower than LC30 after incubation for the time indicated. All experiments were conducted three times and the representative results were presented.

### Animal treatment and sample collection

Experiments were performed in AAALAC accredited facilities at Rutgers, The State University of New Jersey using protocols approved by our Institutional Animal Care and Use Committee. Female Fisher (F344) rats (Harlan Laboratories) were acclimatized to a powdered ration (Tekland). Rats were maintained on the powdered diet (*i.e*., standard AIN-76A diet containing 0.01 ppm selenium as sodium selenite) for one week. The MSC-enriched diet was produced by admixing *L*-Se-methylselenocysteine (Selenium Technologies) with the control diet to a concentration of 3 ppm selenium. Animals were housed under controlled conditions with a 12 hour light/12 hour dark cycle. Zeitgeber Time 0 (ZT 0) was set at 7 AM (light on); ZT12 was set at 7 PM (light off).

NMU (Sigma) was dissolved in acidified saline (pH 5) to a concentration of 10 mg/ml immediately before injection. For time course studies, 63 female F344 rats (aged 55 ± 2 days, 155–175 g) treated with NMU by a single intra-peritoneal (*i.p*.) injection (50 mg/kg body weight), were randomized to 3 groups (21 rats per group). Groups 1, 2, and 3 were sacrificed at days 0, 2, and 30 post-exposure, respectively. For mechanistic studies, 42 NMU-treated rats were randomized into two groups of 21 animals and maintained on control diet or MSC-enriched diet for 30 days. Twenty-one vehicle controls maintained on standardized diet served as the control group. Three rats per group were sacrificed by CO_2_ asphyxiation every 4 hours over a 24-hour period, beginning at 7 AM (ZT0). All mammary glands at each side of individual rats were carefully dissected, combined into a pool of left or right mammary gland tissue sample. Tissue samples were snap-frozen on dry ice, and then stored at −80°C.

### NAD^+^/NADH quantification

Assays were performed with frozen rat mammary gland samples or cultured cells. Frozen right-side mammary gland samples (~50 mg) from 3 rats per group per time were used for NAD^+^/NADH assay. Tissue samples or cell pellets were homogenized, extracted, and filtered using 10 Kd spin column (BioVison) to remove enzymes that rapidly consume NADH. NAD^+^/NADH ratios were determined using NAD^+^/NADH Quantification Kit™ (BioVision) according to the manufacturer instruction. The NAD^+^/NADH was calculated with the formulation (NADt-NADH)/NADH, where NADt is total of NADH and NAD^+^. Three independent samples per group per time were analyzed in triplicate.

### SIRT1 activity assay

Total protein extracts were prepared from a small piece (~50 mg) of tissue from right-side mammary gland of individual rats sacrificed at ZT12 on the day 30 post-exposure, or from cultured cells with RIPA buffer. SIRT1 deacetylase activity was determined with SIRT1 Fluorimetric Drug Discovery Kits™ (Enzo Life Sciences). Briefly, initial deacetylation rates of SIRT1 were determined at 1 unit human recombinant SIRT1 enzyme, 25 μM deacetylase substrate, and 25 μM NAD^+^ (37°C) in the absence (control) or presence of 10 μl of extracted protein. Fluorescence signal was measured with a microplate reader at 360 nm excitation and 460 nm emission wavelengths. Standard curve was produced with serially diluted-deacetylation standard. Activity was normalized to protein concentration and expressed as deacetylated product (μmol)/protein (μg). Three independent samples per group were analyzed in triplicate.

To determine if NMU and MSC affected SIRT1 activity directly, we also performed SIRT1 activity assay with different concentrations of NMU (0, 0.25, 0.5, 1 mM) or MSC (0, 6.3, 12.5, 25 μM) in the presence of purified human recombinant SIRT1 enzyme but absence of extracted tissue protein *ex vivo*.

### Quantitative real-time RT-PCR

Total RNA was extracted from a small piece (~50 mg) of frozen tissue from right-side mammary glands of rats sacrificed on days 0, 2, and 30 post-exposure to NMU with or without MSC-enriched diet. *Per2* mRNA expression levels were determined using real-time quantitative RT-PCR as described previously [13].

### Chromatin immunoprecipitation (ChIP) assay

Pooled frozen right-side mammary tissue samples from three rats (100 mg mammary gland per rat) per group at each time point were used for ChIP assay. Frozen tissues were pulverized and cross-linked with 1% formaldehyde, followed by quenching with 125 mM glycine. Cell pellets were homogenized with cold cell lysis buffer containing protease inhibitor cocktails I & II (Sigma) and processed using EZ-ChIP™ kits (Millipore). Briefly, nuclear and membrane pellets were lysed and sonicated to chromatin fragments of ~200–1000 base pairs. Cross-linked chromatin was pre-cleared and incubated with anti-acetylated BMAL1 (AcBMAL1) antibody (Santa Cruz Biotech) or anti- acetylated H3K9 (AcH3K9) antibody (Santa Cruz Biotech) at 4°C overnight. Normal rabbit IgG served as negative control and anti-RNA polymerase II as positive control. After protein-DNA cross-links were reversed; resolved, purified DNA was used for PCR. PCR was performed with primers targeting the E-box (CACGTG) (sense, 5′-AGCTGGGCTATAGAGGTGCTGA-3′; anti-sense, 5′-CACCGTCTCTGTGGCACGT-3′) or Exon1 region (sense, 5′- AATCAGCTTTCCAAACTGGTTCC-3′; anti-sense, 5′-TGGAGCAGTCACGTCATCCTT-3′) in the *Per2* promoter. PCR conditions were as follows: 1 cycle at 95°C for 1.5 min, 35 cycles of inactivation at 95°C for 45s, annealing at 60°C for 60s, and extension at 72°C for 60s, and 1 final cycle for extension at 72°C for 5 min. PCR products were resolved by electrophoresis and photographed.

### Statistical analyses

Animal number *N* = 3 was used *in vivo* studies. All experiments were performed in triplicate in *in vitro* studies. Intergroup differences were evaluated using one-way ANOVA, followed by Tukey's *post-hoc* test. We used *alpha* = 0.05 or 0.01 as the level of significance for hypothesis testing.
